# Supplementary material for: Predation and fragmentation portrayed in the statistical structure of prey time series
Source: BMC Ecol. 2009 May 6;9:10. doi: 10.1186/1472-6785-9-10 (PMC2689204; doi:10.1186/1472-6785-9-10)
Supplement: Additional file 2 — Voles and related classes ODDox Documentation. ODDox documentation of the agent-based model (ALMaSS) applied by Hendrichsen et al. The documentation is started by activating main.html. [file 1472-6785-9-10-S2.zip › Vole_ODDox/globals_vars.html]

ALMaSS ODDox: Class Members

- Main Page
- Related Pages
- Classes
- Files

- File List
- File Members

- All
- Functions
- Variables
- Typedefs
- Enumerations
- Enumerator
- Defines

- c
- d
- f
- g
- i
- l
- m
- n
- o
- r
- t
- v
- w

### - c -

- cfg\_CIPEGridOutput\_used
  : VolePopulationManager.cpp
  , Vole\_all.cpp- cfg\_extradispmort
    : VolePopulationManager.cpp- cfg\_fungi\_app\_prop
      : WinterWheat.cpp- cfg\_greg\_app\_prop
        : WinterWheat.cpp- cfg\_herbi\_app\_prop
          : WinterWheat.cpp- cfg\_ins\_app\_prop
            : WinterWheat.cpp- cfg\_pm\_eventfrequency
              : VolePopulationManager.cpp- cfg\_pm\_eventsize
                : VolePopulationManager.cpp- cfg\_productapplicendyear
                  : Vole\_all.cpp- cfg\_productapplicstartyear
                    : Vole\_all.cpp- cfg\_ReallyBigOutput\_used
                      : VolePopulationManager.cpp- cfg\_ReallyBigOutputMonthly\_used
                        : VolePopulationManager.cpp
                        , Vole\_all.cpp- cfg\_RipleysOutput\_used
                          : VolePopulationManager.cpp
                          , Vole\_all.cpp

### - d -

- DailyMortChance
  : Vole\_all.cpp- DaysAtMaxGrowth
    : Vole\_all.cpp

### - f -

- FemaleMovement
  : Vole\_all.cpp- FemaleResourceReq
    : Vole\_all.cpp- FemaleTerritoryRange
      : Vole\_all.cpp- FemaleTerritoryRangeSlope
        : Vole\_all.cpp- FemNoMature
          : Vole\_all.cpp- FHabQualThreshold1
            : Vole\_all.cpp- FHabQualThreshold2
              : Vole\_all.cpp- FHabQualThreshold3
                : Vole\_all.cpp

### - g -

- g\_extradispmort
  : VolePopulationManager.cpp
  , Vole\_all.cpp- g\_landscape\_p
    : farmfuncs.cpp
    , farm.cpp- g\_rand\_uni
      : Vole\_all.cpp- g\_rotation
        : croprotation.cpp
        , croprotation.h- g\_SpeedyDivides
          : Vole\_all.cpp- g\_str
            : PopulationManager.cpp- GrowStopDate
              : Vole\_all.cpp- growthperdayF
                : Vole\_all.cpp- growthperdayM
                  : Vole\_all.cpp

### - i -

- InfanticideChanceByAge
  : Vole\_all.cpp

### - l -

- l\_pest\_enable\_pesticide\_engine
  : Vole\_all.cpp- l\_pest\_insecticide\_amount
    : farmfuncs.cpp- l\_pest\_product\_0\_amount
      : farmfuncs.cpp- l\_pest\_product\_1\_amount
        : farmfuncs.cpp- l\_pest\_productapplic\_period
          : farmfuncs.cpp- l\_pest\_productapplic\_startdate
            : farmfuncs.cpp

### - m -

- MaleMovement
  : Vole\_all.cpp- MaleReproductFinish
    : Vole\_all.cpp- MaleTerritoryRange
      : Vole\_all.cpp- MaleTerritoryRangeSlope
        : Vole\_all.cpp- MaturationChance
          : Vole\_all.cpp- MaxFemaleTerrSize
            : Vole\_all.cpp- MaxMaleTerrSize
              : Vole\_all.cpp- MaxStarvationDays
                : Vole\_all.cpp- MaxWeightF
                  : Vole\_all.cpp- MaxWeightM
                    : Vole\_all.cpp- MHabQualThreshold1
                      : Vole\_all.cpp- MHabQualThreshold2
                        : Vole\_all.cpp- MHabQualThreshold3
                          : Vole\_all.cpp- MinFemaleMovement
                            : Vole\_all.cpp- MinFVoleHabQual
                              : Vole\_all.cpp- MinMaleMovement
                                : Vole\_all.cpp- MinMVoleHabQual
                                  : Vole\_all.cpp- MinReproAgeF
                                    : Vole\_all.cpp- MinReproAgeM
                                      : Vole\_all.cpp- MinReproWeightF
                                        : Vole\_all.cpp- MinReproWeightM
                                          : Vole\_all.cpp- MoveToLessFavourable
                                            : Vole\_all.cpp
                                            , VolePopulationManager.cpp- MutationChance
                                              : GeneticMaterial.cpp

### - n -

- NoFarmTypes
  : croprotation.h

### - o -

- owl\_breed\_day
  : Predators.cpp- owl\_breed\_threshold
    : Predators.cpp- owl\_death\_threshold
      : Predators.cpp- owl\_StartingNo
        : Predators.cpp

### - r -

- ReproTable\_base
  : VolePopulationManager.cpp

### - t -

- TheGestationPeriod
  : Vole\_all.cpp

### - v -

- Vector\_x
  : Vole\_all.cpp- Vector\_y
    : Vole\_all.cpp- VoleHarvestMort
      : Vole\_all.cpp- VoleHerbicicideMort
        : Vole\_all.cpp- VoleInsecticideMort
          : Vole\_all.cpp- VoleMoveInterval
            : Vole\_all.cpp- VolePigGrazingMort
              : Vole\_all.cpp- VoleSoilCultivationMort
                : Vole\_all.cpp- VoleStriglingMort
                  : Vole\_all.cpp

### - w -

- WeanedAge
  : Vole\_all.cpp- WeanedWeight
    : Vole\_all.cpp- weasel\_breed\_day
      : Predators.cpp- weasel\_breed\_threshold
        : Predators.cpp- weasel\_death\_threshold
          : Predators.cpp- weasel\_StartingNo
            : Predators.cpp

---

Generated on Thu Jan 22 14:13:48 2009 for ALMaSS ODDox by 
 1.5.6
